# Endogenous Dach1 in cancer

**DOI:** 10.18632/oncoscience.251

**Published:** 2015-09-23

**Authors:** Kongming Wu, Xun Yuan, Richard Pestell

**Affiliations:** Department of Oncology, Tongji Hospital, Huazhong University of Science and Technology, Wuhan, China

**Keywords:** DACH1, cytokine, prostate cancer, tumor suppressor

The *Dachshund* gene is a key component of the retinal determination gene network in Drosophila eye development. Recent studies have demonstrated an important role for the human Dachshund homologue 1 (DACH1) in tumorigenesis [1]. Mechanistic studies demonstrated that DACH1 regulates its target genes via either directly binding to specific DNA sequences within chromatin or indirectly through forming protein complex with other transcriptional factors, including c-Jun and Smad4. DACH1 restrained proliferation, migration *in vitro* and repressed tumor growth and metastasis *in vivo*. There prior observations were based on cultured cells engineering DACH1 expression. The physiological role of the endogenous DACH1 had not been determined in part because genetic deletion in the mice was periembryonic lethal. A recent study report in the May 15 issue of Cancer Research determined the role of endogenous Dach1 in the prostate using tissue specific deletion mice [2].

Using prostate cancer cell lines of distinct genetic background, DACH1 was shown to inhibit proliferation *in vitro* and tumor growth *in vivo* of both AR negative cancer cell (PC-3) and castration resistant AR positive cell (C4-2 and 22RV-1). Consistent with the prior finding that DACH1 inhibited cell cycle progression though inhibiting Cyclin D1 expression, conditional Dach1 knock out in the prostate by using Dach1 ^fl/fl^/Probasin-Cre bi-transgenic increased the expression of cyclin D1, E and A, accompanied by enhanced DNA synthesis and reduced apoptosis [2]. Whole genomic expression profiling with functional pathway analysis identified the cytokine-cytokine receptor interaction as a key target of endogenous Dach1. CXCL family member CXCL-1, 2, 5, 6 and IL-6, 8 expressions were inhibited by DACH1 in PC-3 cell. DACH1 mRNA expression was reduced in metastatic human prostate cancer [3] and DACH1 abundance was inversely correlated with IL-6 and IL-8 (Fig. 1). In agreement, there was 1000 fold activation of IL-6 and IL-8 secretion when endogenous Dach1 was deleted *in vivo* from prostate epithelial cells (PEC). Functional assays, using immune neutralizing antibodies or purified recombinant cytokines to conditioned medium from wt or Dach1 KO PEC, proved that IL-6 and KC (homolog of IL-8 in mice) were the key downstream targets of Dach1 in governing cell migration. In addition, various single oncogene (c-Myc, NeuT, H-Ras or v-Src) transformed prostate epithelial cells (PEC) reduced Dach1 expression both in cultured cells and in extirpated tumor tissues [2]. Combined with previous finding that DACH1 represses ligand induced transcriptional activation of the androgen receptor and prostate cancer cellular proliferation in tissue culture [3], it is likely that DACH1 conveys distinct functions at different stages of prostate cancer onset and progression.

**Figure 1:**
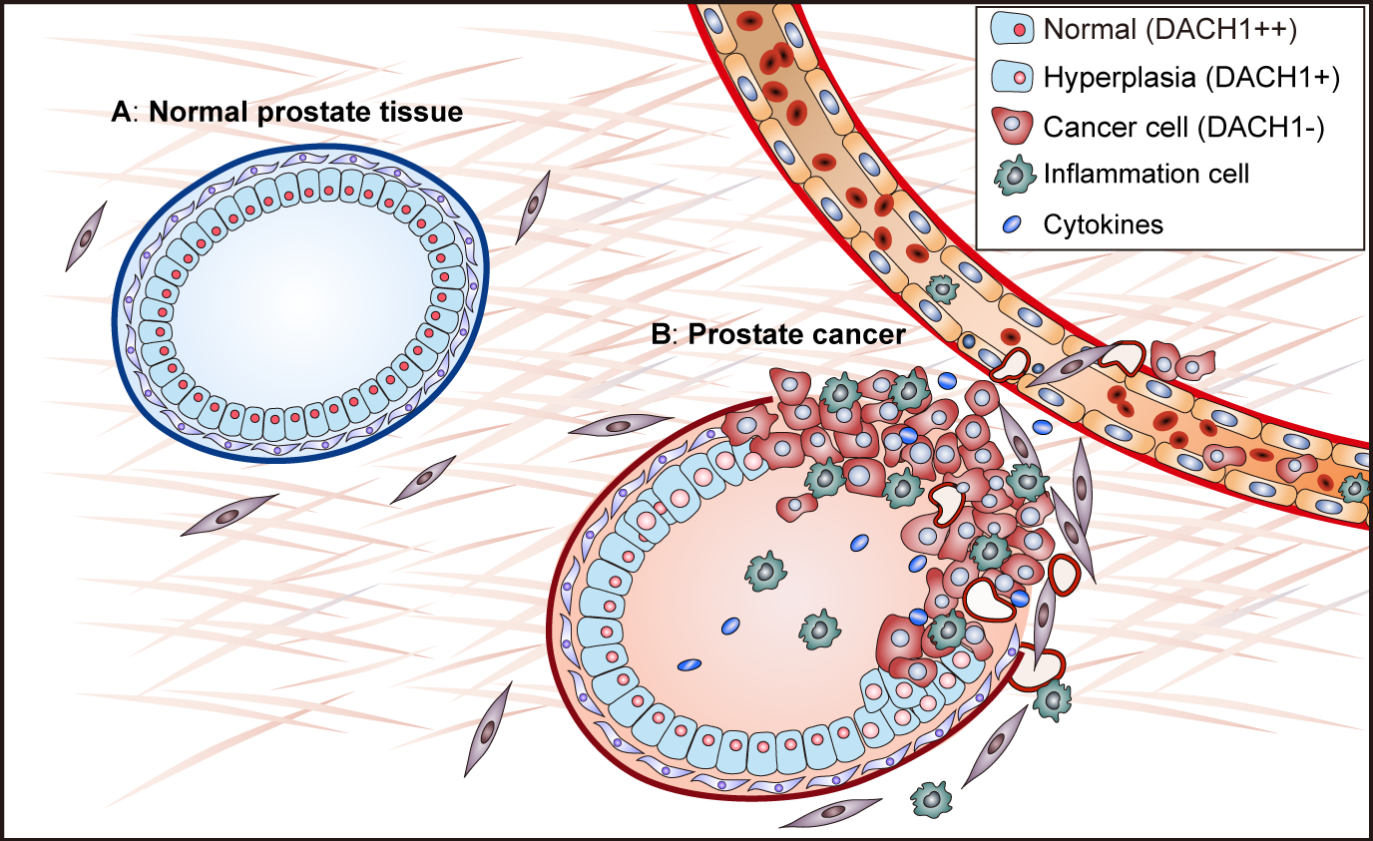
Reduced expression of DACH1 activates heterotypic signaling in prostate cancer

The transition from hormone-dependent to castrate-resistant prostate cancer (CRPC) is the major cause of therapeutic failure. Clinical studies have shown that increased IL-6 and IL-8 signaling correlates with CRPC and predicts poor prognosis [4]. IL-6 and IL-8 produced by prostate cancer cell promote cancer cell proliferation and invasion.

Moreover, IL-8 signaling from tumor cells initiates cancer cell-stromal interaction to induce treatment resistance and angiogenesis [5]. The finding that DACH1 is a key endogenous gene that restrains cytokine signaling may have therapeutic relevance. The finding that DNA demethylation agents could restore DACH1 expression in PC-3 cells [2] suggests targeting DACH1 may be practical.

DACH1 also directly suppressed IL-8 in breast cancer cells and inhibited KC-mediated lung metastasis [6]. A subsequent study in lung cancer has also demonstrated that DACH1 can suppress the secretion of CXCL5, thereby reducing CXCL5-mediated proliferation, migration and invasion. Moreover, as with prostate cancer, a reverse relationship between DACH1 and CXCL5 was reported in tumor samples and low DACH1 correlated with reduced survival in lung cancer patients [7]. Thus, DACH1 governs cell fate through intracellular transcriptional regulation and also through heterotypic signaling that determines cancer-stromal interaction, a key hallmark of cancer [8].

## References

[1] Liu Y (2015). Int J Cancer.

[2] Chen K (2015). Cancer Res.

[3] Wu K (2009). Cancer Res.

[4] Sharma J (2014). Prostate.

[5] Maxwell PJ (2014). Oncotarget.

[6] Wu K (2008). Proc Natl Acad Sci U S A.

[7] Han N (2015). Oncotarget.

[8] Hanahan D (2011). Cell.

